# Supplementary material for: Compact Optical Reservoir Computing via Luminescence Dynamics in Rare‐Earth Ions‐Doped Nanocrystals
Source: Adv Sci (Weinh). 2025 Nov 21;13(7):e17334. doi: 10.1002/advs.202517334 (PMC12866774; doi:10.1002/advs.202517334)
Supplement: Supplementary file 1 — Supporting Information [file ADVS-13-e17334-s001.docx]

**Supplementary** **Information for**

**Compact Optical Reservoir Computing *via* Luminescence Dynamics in Rare-earth Ions-doped Nanocrystals**

Junyan Chen^1,†^, Jingsong Fu^1,†^, Jie Xu^1^, Yixiang Qin^1^, Axin Du^1^, Kaiyang Wang^1,*^, Limin Jin^1,2,*^, Can Huang^1,3,4,*^

^1^ Ministry of Industry and Information Technology Key Lab of Micro-Nano Optoelectronic Information System, Guangdong Provincial Key Laboratory of Semiconductor Optoelectronic Materials and Intelligent Photonic Systems, Harbin Institute of Technology, Shenzhen 518055, China.

^2^ National Key Laboratory of Science and Technology on Advanced Composites in Special Environments, Harbin Institute of Technology, Harbin 150080, China.

^3^ Quantum Science Center of Guangdong-Hongkong Macao Greater Bay Area, Shenzhen 518055, China.

^4^ Heilongjiang Provincial Key Laboratory of Advanced Quantum Functional Materials and Sensor devices, Harbin Institute of Technology, Harbin 150001, China

^†^ These authors contribute equally to this work.

*Corresponding author:

Can Huang: [huangcan@hit.edu.cn](mailto:huangcan@hit.edu.cn);

Limin Jin: [jinlimin@hit.edu.cn](mailto:jinlimin@hit.edu.cn);

Kaiyang Wang: [wangkaiyang@hit.edu.cn](mailto:wangkaiyang@hit.edu.cn).

**Context**

[Note-1. Materials Characterization 3](#_Toc16288)

[Note-2. Optical Setup 7](#_Toc27753)

[Note-3. Kinetic model for the Re^3+^-doped materials 9](#_Toc11891)

[Note-4. Discussion about Re^3+^-Based reservoir network 12](#_Toc8018)

[Note-5. Comparison between Re^3+^ UCNCs Based reservoir network and other physical RCs 18](#_Toc14126)

## **Note-1. Materials Characterization**

**Reagents:** Ytterbium(III) acetate hydrate (99.9%), Yttrium(III) acetate hydrate (99.9%), Gadolinium(III) acetate hydrate (99.9%), Thulium(III) acetate hydrate (99.9%), Sodium hydroxide, Ammonium fluoride, 1-Octadecene, and Oleic acid. All acetates were purchased from Sigma-Aldrich without any further purification.

**Synthesis of NaYF_4_:Gd(10 mol%) Core Nanoparticles:** A mixture of 3.6 mL Y(CH_3_CO_2_)_3_ aqueous solution (0.2 M) and 0.4 mL Gd(CH_3_CO_2_)_3_ aqueous solution (0.2 M) was added to a 100 mL flask containing 6 mL oleic acid (OA) and 14 mL 1-octadecene (ODE). The mixture was heated to 160°C and maintained for 60 minutes to form lanthanide-oleate precursor complexes, then cooled to room temperature naturally. Subsequently, a methanol solution containing NaOH (2 mmol) and NH_4_F (3.2 mmol) was added under stirring at 50°C for 30 minutes. The temperature was then raised to 100°C under vacuum for 30 minutes to remove methanol. Under argon flow, the solution was heated to 300°C and maintained for 1 hour before cooling to room temperature. The nanoparticles were precipitated by ethanol addition, collected by centrifugation at 6000 rpm for 3 minutes, washed several times with ethanol, and finally dispersed in cyclohexane for further use.

**Synthesis of NaYF_4_:Gd(10 mol%)@ NaYbF_4_:Tm(5 mol%) Core-Shell Nanoparticles:** A 50 mL flask was charged with 2 mL of corresponding lanthanide acetate aqueous solution (0.2 M), 3 mL OA, and 7 mL 1-ODE. The mixture was heated to 160°C for 40 minutes to form lanthanide-oleate precursors, then cooled to room temperature. A methanol solution of NaOH (1 mmol) and NH_4_F(1.6 mmol) was added and stirred at 50°C for 30 minutes, followed by vacuum heating at 100°C for 30 minutes to remove methanol. For shell growth, pre-synthesized core nanoparticles (0.6 mmol) were mixed with 3 mL OA and 7 mL ODE, heated to 300°C under argon, then injected with shell precursor solution and maintained for 1 hour. The resulting nanoparticles were collected by ethanol precipitation, centrifuged at 6000 rpm for 3 minutes, washed with ethanol, and redispersed in cyclohexane. Shell thickness was controlled by varying the precursor injection volume.

**Synthesis of NaYF_4_:Gd(10 mol%)@NaYbF_4_:Tm(5 mol%)@NaYF_4_ Core-Shell-Shell Nanoparticles:** Following the same procedure as core-shell synthesis, except using preformed core-shell nanoparticles as seeds. The shell precursor solution (2 mL of 0.2 M lanthanide acetate with 3 mL OA and 7 mL 1-ODE) was prepared similarly. After heating to 300°C under argon, the precursor was injected to the core-shell nanoparticle solution and maintained for 1 hour. Final nanoparticles were collected by ethanol precipitation, centrifugation (6000 rpm, 3 minutes), ethanol washing, and cyclohexane dispersion.

**ii. Characterization:** The morphology of the nanoparticles was carried out on a FEI/Philips Tecnai 12 BioTWIN transmission electron microscope operating at an acceleration voltage of 120 kV. High-resolution TEM was performed on a JEOL-JEM 2100F transmission electron microscope operating at an acceleration voltage of 200 kV. Scanning electron microscopy (SEM) image of the film were measured by a Hitachi Model S-4700 scanning electron microscope with an accelerating voltage of 10-30 kV. Powder X-ray diffraction (XRD) data were recorded on a Bruker AXS D2 phaser with a graphite-monochro-matized Cu Kα radiation (1.5406 Å). Unless otherwise stated, all measurements were carried out at room temperature.

| 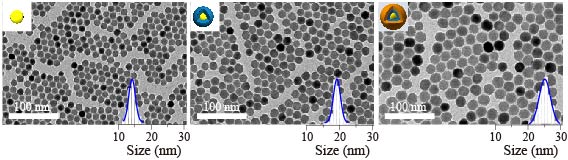 |
| --- |
| Figure S1. TEM images of the (a) core, (b) core-shell, and (c) core-shell-shell NaYF_4_:Gd(10 mol%)@NaYbF_4_:Tm(5 mol%)@NaYF_4_ nanoparticles. The scales bars are 100 nm. The corresponding size distributions of the nanoparticles are attached as insets. |

| 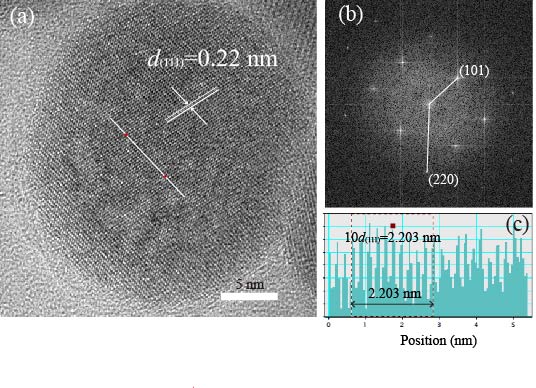 |
| --- |
| Figure S2. Analysis of crystal lattice in the core-shell-shell nanocrystal. (a) High-resolution TEM image. The scale bar is 5 nm. (b) FFT pattern of the whole particle in (a). (c) Intensity profile recorded by scanning along the line shown in (a), revealing a d-spacing of 0.22 nm. |

| 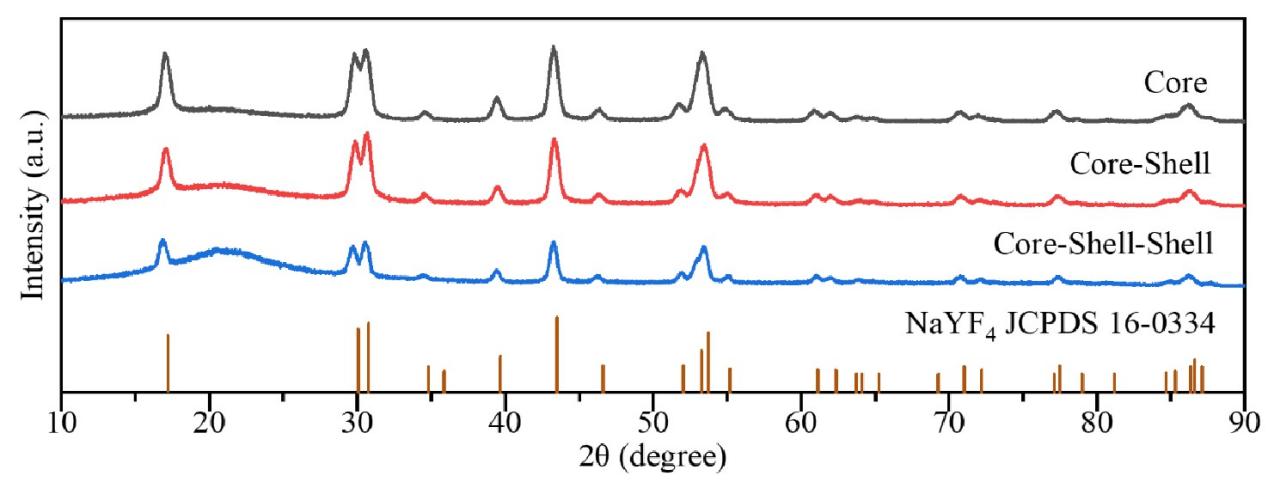 |
| --- |
| Figure S3. XRD patters of the core, core-shell, and core-shell-shell nanoparticles. The line spectrum of hexagonal phase NaYF_4_ crystal (JCPDS #16-0334) is also attached for the reference. The results show pure hexagonal phase of the products. |

| 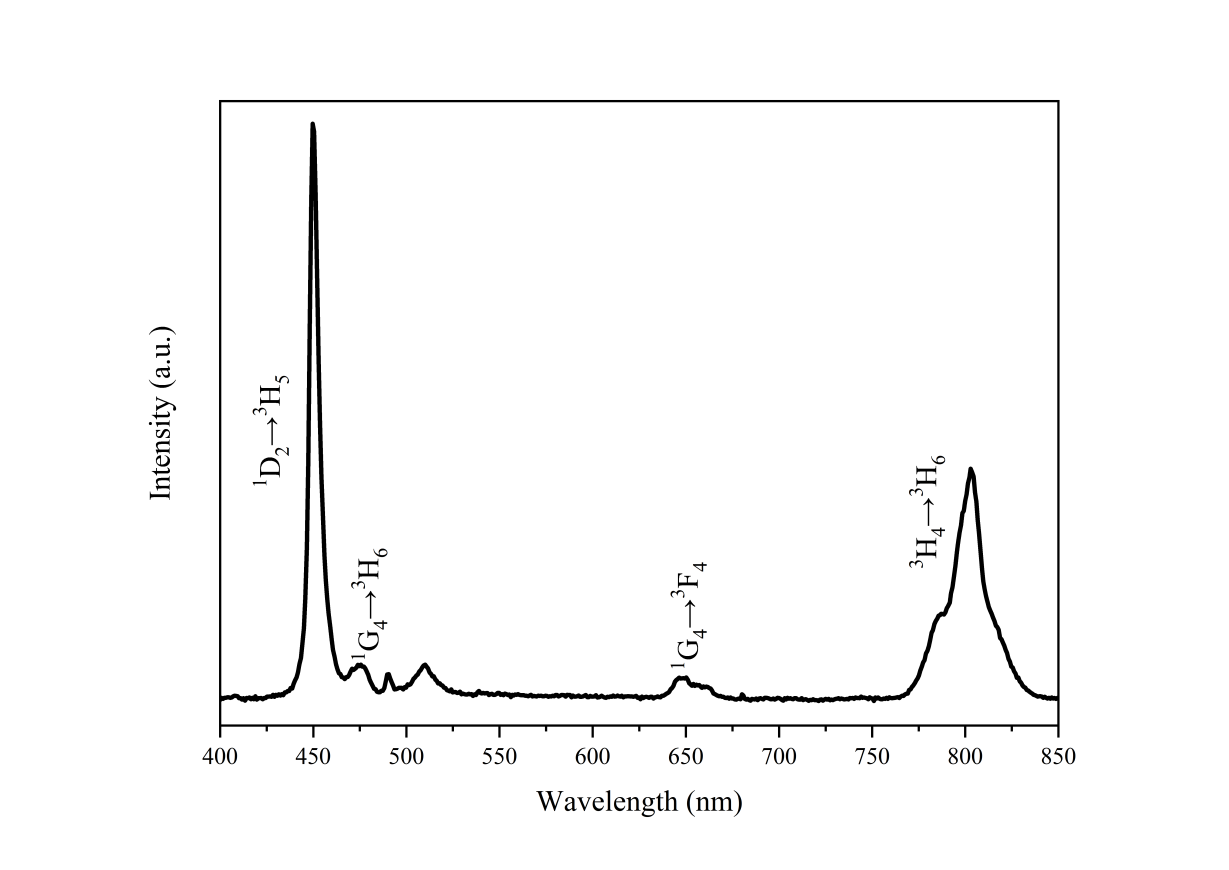 |
| --- |
| Figure S4. The whole pattern of the as-synthesized core-shell-shell nanoparticles under 980 nm excitation (3.58 kW/cm^2^). |

## **Note-2. Optical Setup**

To measure the dynamical response of the Re^3+^ film, we conducted luminescence characterization of the samples using the optical setup illustrated in Figure S5. The 980 nm continuous-wave laser (MDL-H-980-2W, CNI)) using an external modulator to achieve different pump intensities, pulse widths, and pulse intervals. The modulation module consists of a lithium niobate electro-optic modulator (EOM, EO-AM-R-20-C1, Thorlabs) and two orthogonal linear polarizers. The signal from an arbitrary waveform generator (AWG; UTG9604T, UNI-T) was amplified by an Radio Frequency amplifier (RFA) before being applied to the EOM. The signal light is focused onto the rare-earth thin film through a 50x objective lens. The fluorescence emitted by the rare-earth material is collected by the objective lens, with one portion captured by a spectrometer (QE65pro, Ocean Optics/UpC-AS, Biaoqi Optoelectronics) and another portion detected by an avalanche photodetector(APD, 2051-FS-M, Newport) to measure the luminescence intensity. The signal from the APD was acquired/recorded by a digital oscilloscope (MSO3054X, UNI-T). The whole optical path length Iis around 100 cm and system footprint is around 100 × 10 cm².


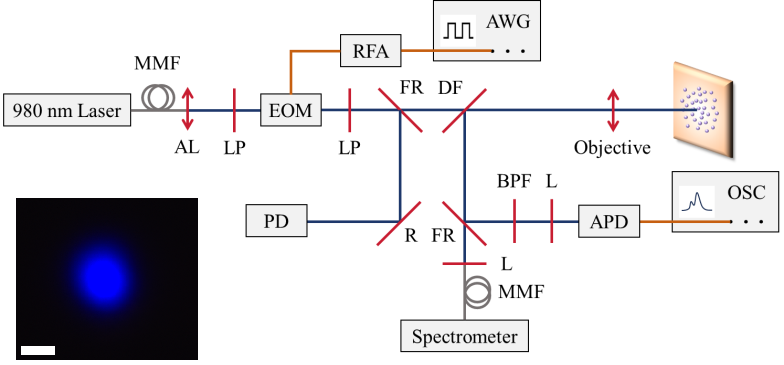


Figure S5. Optical setup for measuring dynamical response of Re^3+^ nanocrystal film. The inset shows the optical image of the sample under 980 nm pumping. Scale bar: 20 μm.

The lifetime measurement was performed on Horiba by monitoring the upshifting emission under the excitation of 980 nm laser. The effective lifetimes were fitted by

$\text{y}\text{=}\text{A}_{\text{1}}\text{ⅇ}^{\text{-}\text{x}/{\text{τ}_{\text{1}}}}\text{+}\text{A}_{\text{2}}\text{ⅇ}^{\text{-}\text{x}/{\text{τ}_{\text{2}}}}\text{+}\text{y}_{\text{0}}$ (2-1)

$\text{τ}\text{=}\frac{\text{A}_{\text{1}}\text{τ}_{\text{1}}^{\text{2}}\text{+}\text{A}_{\text{2}}\text{τ}_{\text{2}}^{\text{2}}}{\text{A}_{\text{1}}\text{τ}_{\text{1}}\text{+}\text{A}_{\text{2}}\text{τ}_{\text{2}}}$ (2-2)

| 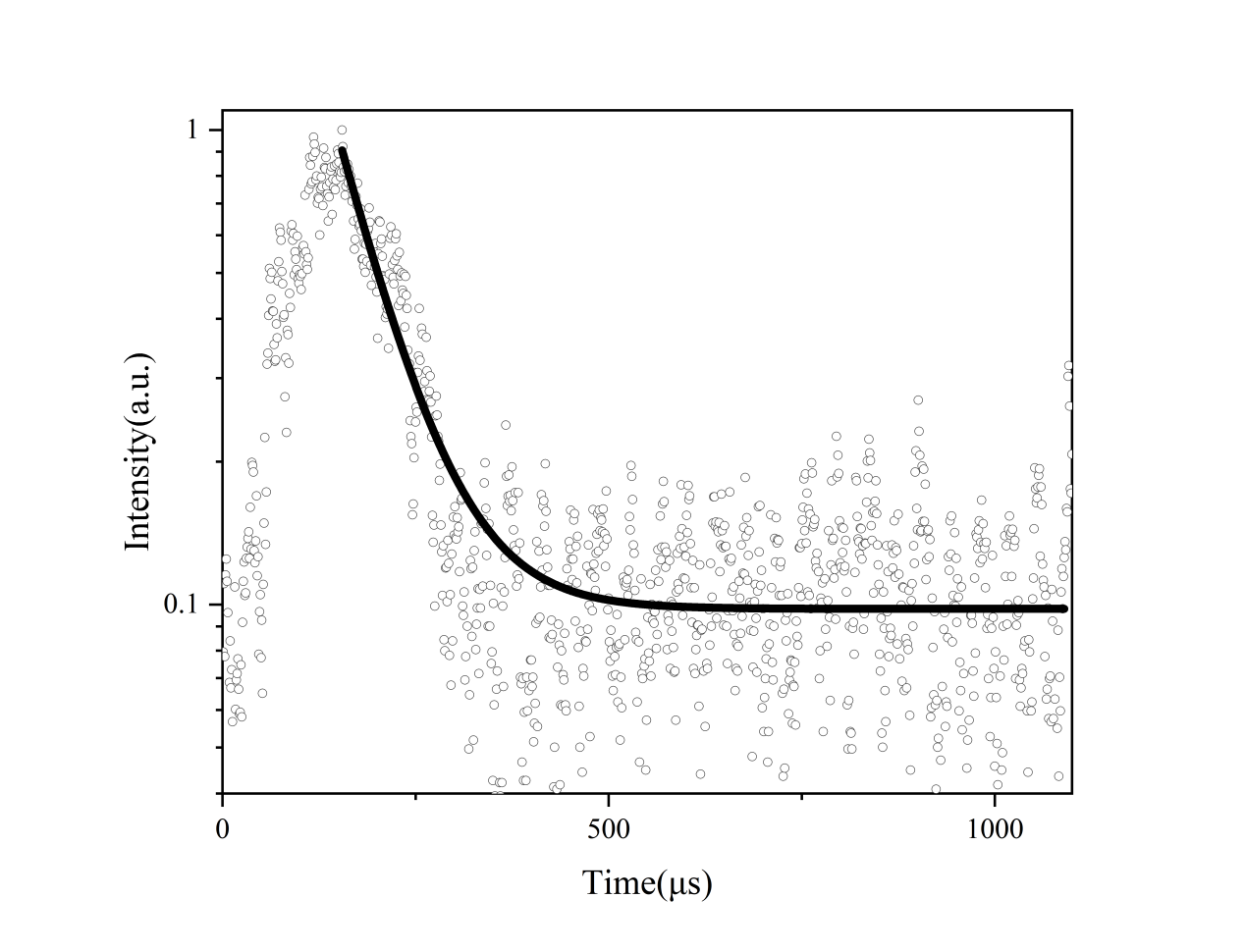 |
| --- |
| Figure S6. Time-dependent emission spectrum of Tm^3+^ ions monitored at 451 nm (65 μs) under 980 nm excitation. Note that the solid line is intended to guide the eyes. |

In the main text, we systematically investigated the influence of pulse width, pulse number, and pulse interval on the luminescence dynamics under continuous multi-pulse excitation. The signals loaded onto the AWG are shown in the figure S7 below, and the corresponding outputs are presented in Figures 3d-f of the main text.


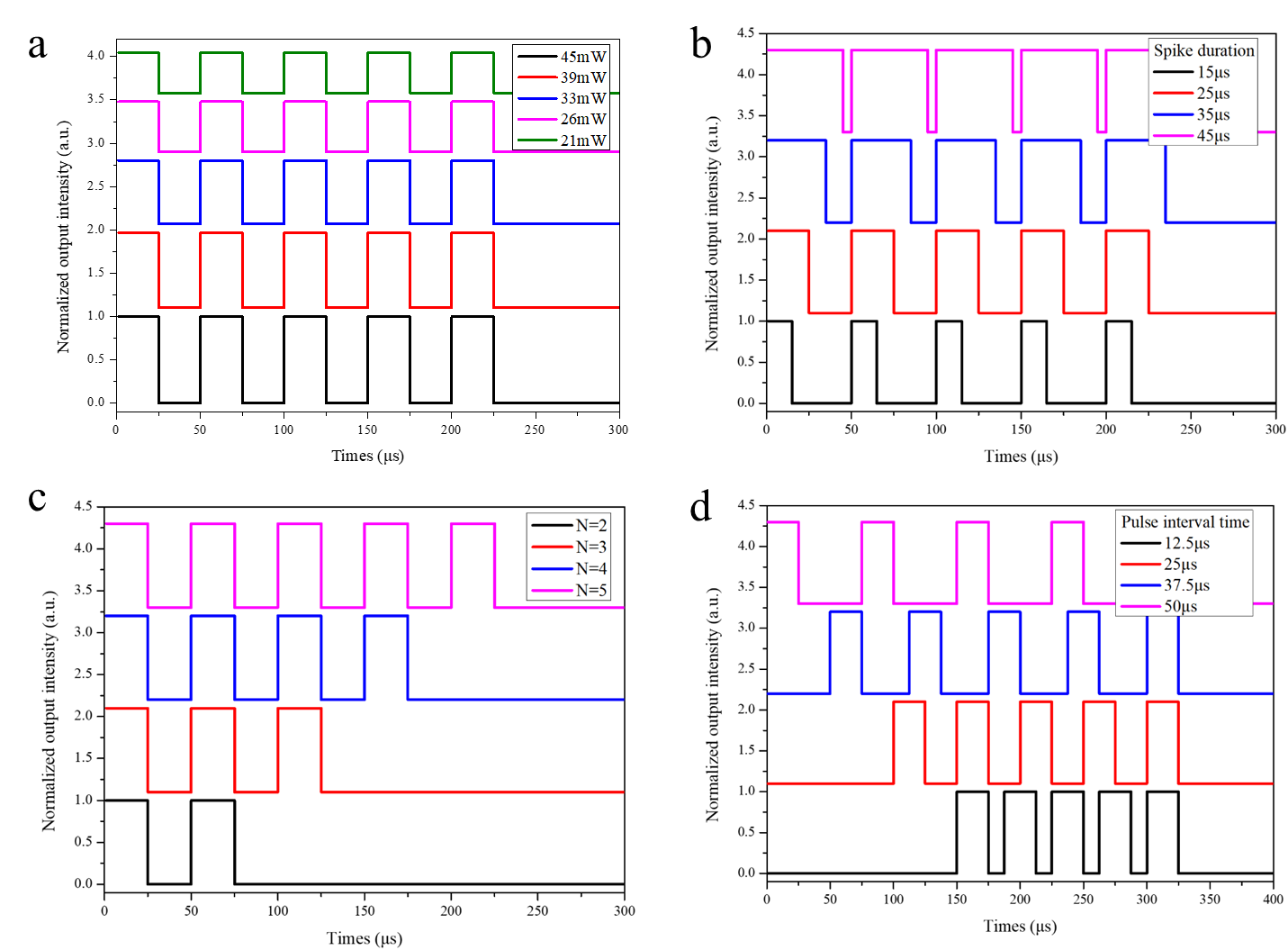


Figure S7. The signals loaded onto the AWG for investigating d the influence of pulse width, pulse number, and pulse interval on the luminescence dynamics.

## **Note-3. Kinetic model for the Re^3+^-doped materials**

In the main text (Fig. 2), we provide a comprehensive discussion on the nonlinear luminescence response characteristics of rare-earth ions. Furthermore, in this section, we present a more quantitative description of the luminescence response through the rare-earth rate equation system, where specific parameters are determined to accurately match our material's response characteristics. The dynamic processes can be quantitatively described by the following rate equations:

$\left\{ \begin{aligned} \frac{\text{d}\text{n}_{\text{s}_{\text{1}}}}{\text{dt}}\text{=-}\text{P}_{\text{in}}\text{n}_{\text{s}_{\text{1}}}\text{+}\text{W}_{\text{s}}\text{n}_{\text{s}_{\text{2}}}\text{+(}\text{c}_{\text{1}}\text{n}_{\text{1}}\text{+}\text{c}_{\text{2}}\text{n}_{\text{2}}\text{+}\text{c}_{\text{3}}\text{n}_{\text{3}}\text{+}\text{c}_{\text{4}}\text{n}_{\text{4}}\text{)}\text{n}_{\text{s}_{\text{2}}} \\ \frac{\text{d}\text{n}_{\text{s}_{\text{2}}}}{\text{dt}}\text{=}\text{P}_{\text{in}}\text{n}_{\text{s}_{\text{1}}}\text{-}\text{W}_{\text{s}}\text{n}_{\text{s}_{\text{2}}}\text{-(}\text{c}_{\text{1}}\text{n}_{\text{1}}\text{+}\text{c}_{\text{2}}\text{n}_{\text{2}}\text{+}\text{c}_{\text{3}}\text{n}_{\text{3}}\text{+}\text{c}_{\text{4}}\text{n}_{\text{4}}\text{)}\text{n}_{\text{s}_{\text{2}}} \end{aligned} \right.$ (3-1)

$\left\{ \begin{aligned} \frac{\text{d}\text{n}_{\text{1}}}{\text{dt}}\text{=(-}\text{c}_{\text{1}}\text{n}_{\text{s}_{\text{2}}}\text{n}_{\text{1}}\text{)+(}\text{b}_{\text{21}}\text{W}_{\text{2}}\text{n}_{\text{2}}\text{+}\text{b}_{\text{31}}\text{W}_{\text{3}}\text{n}_{\text{3}}\text{+}\text{b}_{\text{41}}\text{W}_{\text{4}}\text{n}_{\text{4}}\text{+}\text{b}_{\text{51}}\text{W}_{\text{5}}\text{n}_{\text{5}}\text{)-(}\text{k}_{\text{1}}\text{n}_{\text{1}}\text{n}_{\text{3}}\text{+}\text{k}_{\text{2}}\text{n}_{\text{1}}\text{n}_{\text{4}}\text{+}\text{k}_{\text{3}}\text{n}_{\text{1}}\text{n}_{\text{5}}\text{)} \\ \frac{\text{d}\text{n}_{\text{2}}}{\text{dt}}\text{=}\left( \text{c}_{\text{1}}\text{n}_{\text{s}_{\text{2}}}\text{n}_{\text{1}}\text{-}\text{c}_{\text{2}}\text{n}_{\text{s}_{\text{2}}}\text{n}_{\text{2}} \right)\text{+(-}\text{W}_{\text{2}}\text{n}_{\text{2}}\text{+}\text{b}_{\text{32}}\text{W}_{\text{3}}\text{n}_{\text{3}}\text{+}\text{b}_{\text{42}}\text{W}_{\text{4}}\text{n}_{\text{4}}\text{+}\text{b}_{\text{52}}\text{W}_{\text{5}}\text{n}_{\text{5}}\text{)+(2}\text{k}_{\text{1}}\text{n}_{\text{1}}\text{n}_{\text{3}}\text{+}\text{k}_{\text{2}}\text{n}_{\text{1}}\text{n}_{\text{4}}\text{)} \\ \frac{\text{d}\text{n}_{\text{3}}}{\text{dt}}\text{=}\left( \text{c}_{\text{2}}\text{n}_{\text{s}_{\text{2}}}\text{n}_{\text{2}}\text{-}\text{c}_{\text{3}}\text{n}_{\text{s}_{\text{2}}}\text{n}_{\text{3}} \right)\text{+}\left( \text{-}\text{W}_{\text{3}}\text{n}_{\text{3}}\text{+}\text{b}_{\text{43}}\text{W}_{\text{4}}\text{n}_{\text{4}}\text{+}\text{b}_{\text{53}}\text{W}_{\text{5}}\text{n}_{\text{5}} \right)\text{+(-}\text{k}_{\text{1}}\text{n}_{\text{1}}\text{n}_{\text{3}}\text{+}\text{k}_{\text{2}}\text{n}_{\text{1}}\text{n}_{\text{4}}\text{+2}\text{k}_{\text{3}}\text{n}_{\text{1}}\text{n}_{\text{5}}\text{)} \\ \frac{\text{d}\text{n}_{\text{4}}}{\text{dt}}\text{=}\left( \text{c}_{\text{3}}\text{n}_{\text{s}_{\text{2}}}\text{n}_{\text{3}}\text{-}\text{c}_{\text{4}}\text{n}_{\text{s}_{\text{2}}}\text{n}_{\text{4}} \right)\text{+}\left( \text{-}\text{W}_{\text{4}}\text{n}_{\text{4}}\text{+}\text{b}_{\text{54}}\text{W}_{\text{5}}\text{n}_{\text{5}} \right)\text{+(-}\text{k}_{\text{2}}\text{n}_{\text{1}}\text{n}_{\text{4}}\text{)} \\ \frac{\text{d}\text{n}_{\text{5}}}{\text{dt}}\text{=}\left( \text{c}_{\text{4}}\text{n}_{\text{s}_{\text{2}}}\text{n}_{\text{4}} \right)\text{+}\left( \text{-}\text{W}_{\text{5}}\text{n}_{\text{5}} \right)\text{+(-}\text{k}_{\text{3}}\text{n}_{\text{1}}\text{n}_{\text{5}}\text{)} \end{aligned} \right.$ (3-2)

The multi-level dynamics of Yb³⁺/Tm³⁺ rare-earth ions are illustrated in the figure 1b. Here, we assume rapid non-radiative transitions between ³F₂→³H₄, ³F₃→³H₄, and ³H₅→³F₄, thereby simplifying the system to a 2+5-level dynamic process, as shown in Figure S8. Equation (3-1) describes the level dynamics of Yb³⁺, where $\text{n}_{\text{s}_{\text{i}}}$ represent the population densities at energy levels${\text{ }\text{s}}_{\text{i}}$ in Yb³⁺, and $\text{n}_{\text{j}}$ represent the population densities at energy levels $\text{j}$ in Tm³⁺. The term $\text{P}_{\text{in}}$ denotes the stimulated absorption rate of Yb³⁺ ions under optical pumping, which is proportional to the energy density of the pump beam. The parameter $\text{c}_{\text{i}}$ characterizes the energy transfer upconversion (ETU) process from $\text{s}_{\text{2}}$ to $\text{n}_{\text{i}\text{+1}}$ , while $\text{b}_{\text{jk}}\text{W}_{\text{j}}$ describes the transition process from energy level $\text{j}$ to $\text{k}$. The terms $\text{k}_{\text{1}}\text{, }\text{k}_{\text{2}}\text{, }\text{k}_{\text{3}}$ correspond to the three possible cross-relaxation (CR) processes. Notably, both ETU and CR processes are essentially non-radiative energy transfer mechanisms between neighboring ions, influenced by interionic distance, spectral overlap, and doping concentration. Consequently, these processes are affected by multiple energy level populations and appear as quadratic terms in the rate equation model. The model neglects transition processes involving defect levels, and for any given condition, the following holds:

$\sum_{\text{k}} \text{b}_{\text{jk}}\text{W}_{\text{j}}\text{=1}$ (3-3)

It should be noted that Equation (3-3) constitutes an overdetermined system of equations. By summing the two equations respectively, we obtain:

$\left\{ \begin{aligned} \frac{\text{d}\text{n}_{\text{s}_{\text{1}}}}{\text{dt}}\text{+}\frac{\text{d}\text{n}_{\text{s}_{\text{2}}}}{\text{dt}}\text{=0} \\ \frac{\text{d}\text{n}_{\text{1}}}{\text{dt}}\text{+}\frac{\text{d}\text{n}_{\text{2}}}{\text{dt}}\text{+}\frac{\text{d}\text{n}_{\text{3}}}{\text{dt}}\text{+}\frac{\text{d}\text{n}_{\text{4}}}{\text{dt}}\text{+}\frac{\text{d}\text{n}_{\text{5}}}{\text{dt}}\text{=0} \end{aligned} \right.$ (3-4)

Normalizing Equation (3-4), we obtain:

$\left\{ \begin{aligned} \text{n}_{\text{s}_{\text{1}}}\text{+}\text{n}_{\text{s}_{\text{2}}}\text{=1} \\ \text{n}_{\text{1}}\text{+}\text{n}_{\text{2}}\text{+}\text{n}_{\text{3}}\text{+}\text{n}_{\text{4}}\text{+}\text{n}_{\text{5}}\text{=1} \end{aligned} \right.$ (3-5)

The parameters in the rate equations are listed in Tables 1. The rate equations were numerically solved using the fourth-order Runge-Kutta method, with simulation results presented in the corresponding figures. The simulated output at 451 nm is shown in the main-text figures, demonstrating excellent agreement with experimental data that validates the rationality of our rate equation model. It should be noted that while our model has comprehensively considered various influencing factors including energy level transitions, cross-relaxation processes, and transition branching ratios during parameter selection and system simplification [1,2], it does not fully account for certain effects such as: (i) all multi-phonon non-radiative processes in Yb^3+^ upconversion, (ii) high-order coupling between different energy levels, and (iii) near-field quenching effects at extremely high concentrations. Nevertheless, the numerical simulations based on these rate equations show remarkable consistency with experimental measurements in temporal evolution dynamics (see Figs. 2c and 5b in the main text), confirming that the model successfully captures the primary kinetic characteristics of the system. This establishes a reliable physical foundation for further in-depth investigations.

| spontaneous radiation coefficients (s^-1^) | | upconversion coefficients | | branching ratios | | | | cross-relaxation coefficients | |
| --- | --- | --- | --- | --- | --- | --- | --- | --- | --- |
| $\text{W}_{\text{s}}$ | 7.6$\text{×}$10^3^ | $\text{c}_{\text{1}}$ | 6.2$\text{×}$10^4^ | $\text{b}_{\text{21}}$ | 1 | $\text{b}_{\text{43}}$ | 0.58 | $\text{k}_{\text{1}}$ | 1.5$\text{×}$10^5^ |
| $\text{W}_{\text{2}}$ | 6.4$\text{×}$10^3^ | $\text{c}_{\text{2}}$ | 5.7$\text{×}$10^4^ | $\text{b}_{\text{31}}$ | 0.27 | $\text{b}_{\text{51}}$ | 0.24 | $\text{k}_{\text{2}}$ | 1.8$\text{×}$10^5^ |
| $\text{W}_{\text{3}}$ | 1.8$\text{×}$10^4^ | $\text{c}_{\text{3}}$ | 7.4$\text{×}$10^4^ | $\text{b}_{\text{32}}$ | 0.73 | $\text{b}_{\text{52}}$ | 0.23 | $\text{k}_{\text{3}}$ | 4.8$\text{×}$10^4^ |
| $\text{W}_{\text{4}}$ | 1.4$\text{×}$10^4^ | $\text{c}_{\text{4}}$ | 5.2$\text{×}$10^3^ | $\text{b}_{\text{41}}$ | 0.18 | $\text{b}_{\text{53}}$ | 0.20 |  |  |
| $\text{W}_{\text{5}}$ | 1.5$\text{×}$10^4^ |  |  | $\text{b}_{\text{42}}$ | 0.24 | $\text{b}_{\text{54}}$ | 0.33 |  |  |

Table S1. The parameters used for simulate the dynamical response of Re^3+^ film.

Figure S8(b) is a simplified diagram of energy level transitions, including the main pathways that affect the 451 nm and 802 nm emissions. Under low pump power, due to a fast cross-relaxation process (¹D₂→³H₄, ³H₆→³H₄), almost no population is excited to the ¹D₂ level. As a result, the 451 nm emission is suppressed, and the population of the ³H₄ energy level increases. This manifests as the emission intensity at 802 nm being much stronger than that at 451 nm. When the pump energy surpasses the energy loss from cross-relaxation, the 451 nm emission intensifies rapidly. A numerical simulation of this process is shown in the figure S8(c-d). It can be observed that, compared to low-power pumping, the population of n₃ decreases significantly under high-power pumping. Simultaneously, the population of n₅ increases markedly, eventually surpassing n₃. This further corroborates the mechanism behind the 451 nm emission. Figure S8(e) displays the emission intensity ratio between 451 nm and 802 nm. Below a pump intensity of 15 mW, the emission at 451 nm remains weaker than that at 802 nm. However, as the pump intensity exceeds 15 mW, the 451 nm emission surpasses the 802 nm signal, exhibiting a clear cross-over transition. This intensity-dependent spectral behavior quantitatively validates the consistency between numerical simulations and experimental observations.


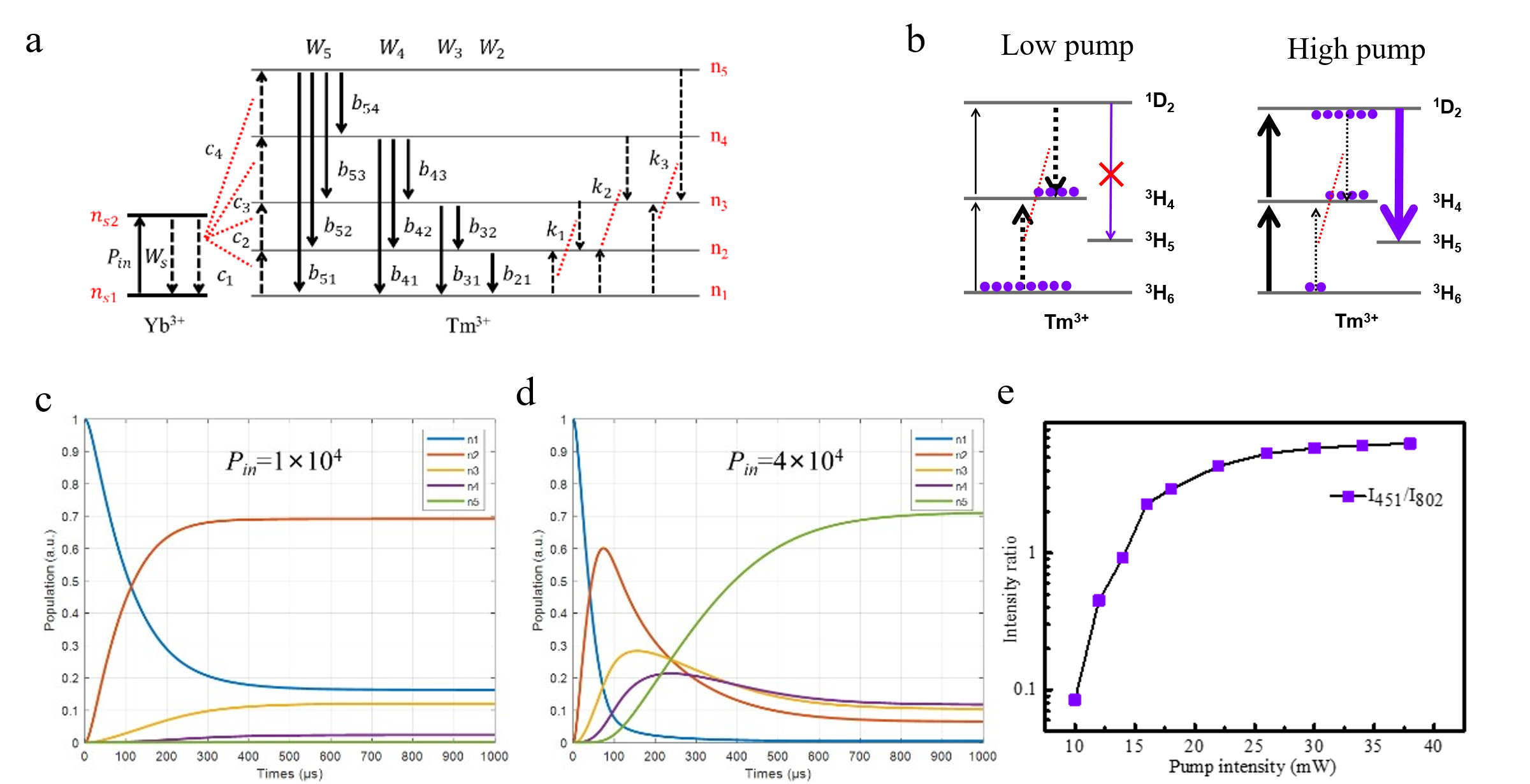


Figure S8. Kinetic model for the Re3+-doped materials. (a) Energy level of Re^3+^ ions. (b) The simplified multi-level dynamics of Yb³⁺/Tm³⁺ rare-earth ions. (c) and (d) show the numerical simulation result for dynamical response of Re^3+^ ions under different pumping densities. (e) Experimental results for emission intensity ratio between 451 nm and 802 nm.

## **Note-4. Discussion about Re^3+^-Based reservoir network**


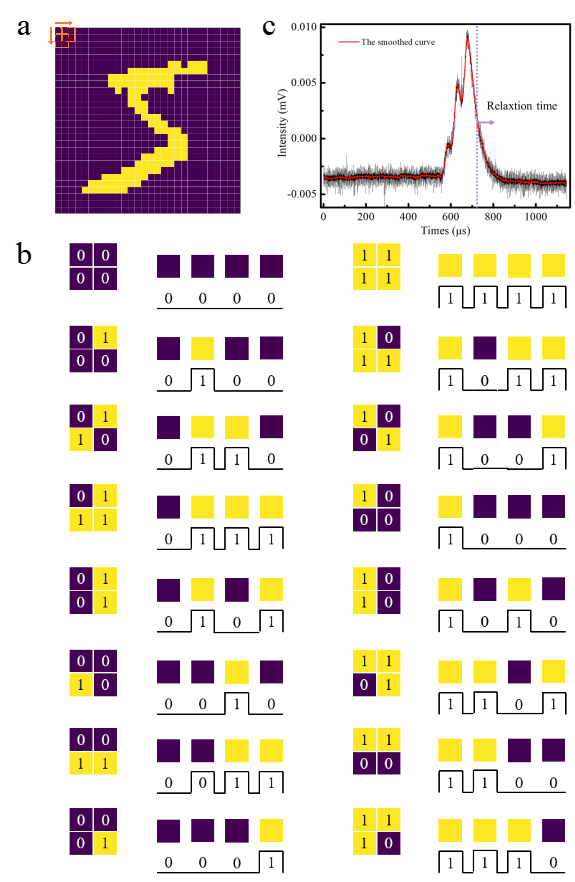


Fig. S9. Image encoding methods. (a) Sliding window format. (b) 4-bit graded neuron encoding format. (c) Typical signal responses captured by the oscilloscope when the input pattern is "1110"

Figure S9(a) elaborately demonstrates the input image encoding mechanism based on the sliding window method. Specifically, the system employs a 2×2 pixel sliding window to traverse the original image in a raster-scan manner (from left to right, top to bottom), converting it into a 4-bit input sequence as shown in Figure S9(b). This binary encoding scheme effectively transforms two-dimensional image data into temporal signal streams suitable for system processing. Figure S9(c) displays typical signal responses captured by the oscilloscope when the input pattern is "1110". The temporal evolution of output signals reveals that as the relaxation time increases, the signal amplitude shows significant attenuation, while the transient response exhibits characteristic exponential decay behavior, confirming the dominant relaxation mechanism governing the system dynamics. Moreover, longer relaxation times can naturally attenuate high-frequency noise components. These characteristics make the method particularly suitable for robust image processing applications requiring noise immunity.

In the main context, we shown that experimental evaluation using the standard MNIST handwritten digit datasets yielded a classification accuracy of 90.7% on 10,000 test images based on the Re^3+^ film RC. Here we present more detailed analysis for the classification task. As shown in figure S10, the average F1-Score value reached approximately 0.91, with digits "0", "1", and "6" demonstrating the best performance. For digit “0”, we observed a recall of 0.9735, precision of 0.9455, yielding an F1-Score of 0.9593. This indicates that the model correctly identified most handwritten zeros (97.35%), while 94.55% of samples predicted as zeros were true positives. Digit 1 achieved even better performance with 0.9665 recall, 0.9581 precision, and a leading F1-Score of 0.9623. Digit 6 also ranked among the top performers with 0.9509 recall, 0.9305 precision, and a 0.9406 F1-Score. The superior performance on these three categories can be attributed to: (1) their relatively standardized stroke patterns and high distinctiveness in the MNIST dataset, and (2) the model's strong capability in capturing horizontal and vertical stroke structures. Overall, the model demonstrated exceptionally low error rates (≤3.5%) for digits 0, 1, and 6, establishing them as robust and highly reliable categories.

In contrast, several digits showed relatively weaker performance, particularly "5", "8", "9", and "2". Digit “5” exhibited the poorest metrics among all categories: only 0.8386 recall (meaning ~16.14% of fives were misclassified as other digits), 0.8842 precision, and an F1-Score of just 0.8608. This suggests significant confusion between 5 and similar-looking digits (particularly 3, 8, and 6), along with an 11.58% false positive rate when the model predicted 5. Digit 8 also showed subpar performance (recall: 0.8676; precision: 0.8614; F1-Score: 0.8645), frequently being confused with “3”, “9”, or “2” in both directions. Digit 9 performed slightly better than “8” (recall: 0.8622; precision: 0.8932; F1-Score: 0.8775) but still fell short of the top categories. For digit 2, while maintaining a decent precision of 0.9126, its recall dropped to 0.8798 (F1-Score: 0.8959), indicating particular confusion with digits “8” and “3”. The common characteristics of these underperforming categories include: (1) complex stroke intersections (especially in “5” and “8”), and (2) greater writing style variations in the MNIST datasets, making it challenging for the model to establish sufficient separation from neighboring categories in the feature space. These findings suggest that the model's performance is closely related to the structural complexity and inter-class similarity of different digits.


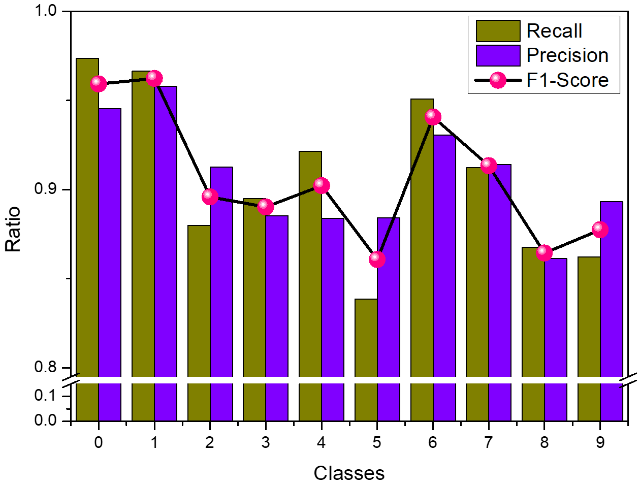


Figure S10. Classification performance for the MNIST handwritten digit datasets.

Quantitative statistics and performance reporting: Repeated over Four independent runs, yielding consistent results (accuracy = 90 ± 1%). The output of rare earth films at four different positions was experimentally measured and compared with Figure 4b in the main text. These results were used to repeat the MNIST classification task.


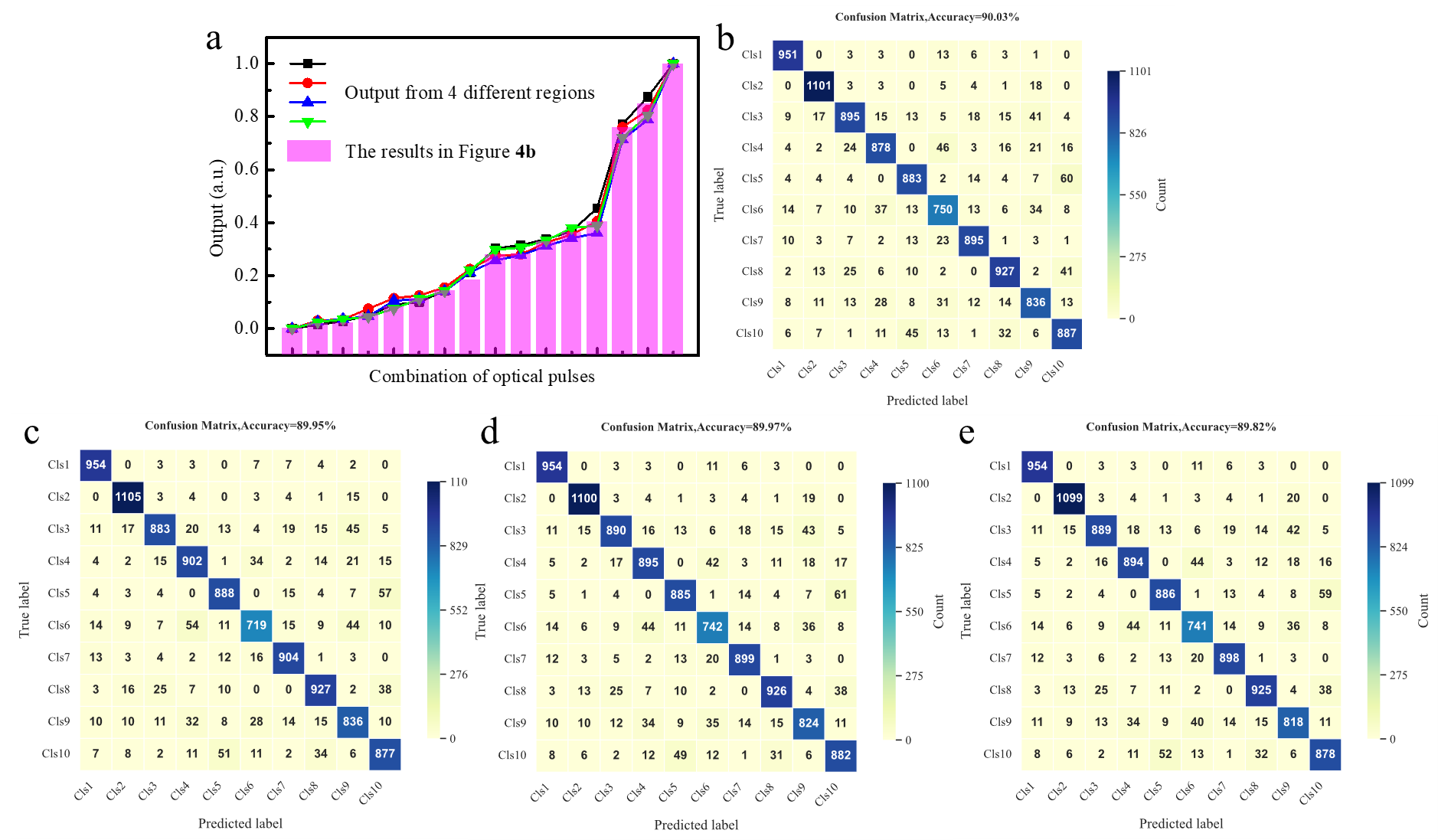


Figure S11. Four independent classification tasks used for the repeatability test.

Undoped‑film control (to exclude detector persistence): We conducted time‑response measurements using an undoped host film (without Tm³⁺ ions) under identical excitation and detection conditions. The undoped film exhibited negligible nonlinear response and no measurable memory effect, confirming that the temporal dynamics observed in the main experiments indeed arise from the UCNCs rather than detector artifacts.

Figure S12. Output response of the un-doped film.

Linear‑path control: A bypass configuration was implemented where the UCNC layer was replaced by a linear optical path (neutral optics only). As expected, this configuration produced a strictly linear input–output relation without fading memory or classification capability, further ruling out trivial mapping behavior.


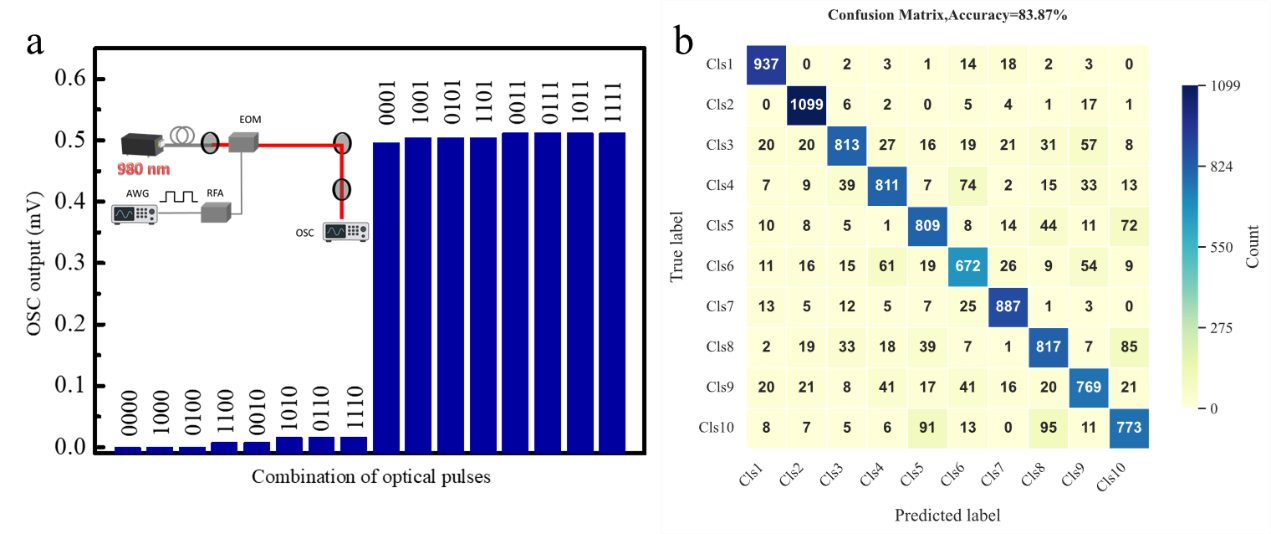


Figure S13. (a) Output response and (b) confusion matrix of the linear system.

Memory-dependent control: We introduced a larger input pulse interval while keeping the same optical intensities and mean values. The reservoir output lost its predictable correlation with the target signal, resulting in a dramatic drop in forecasting performance (Accuracy ~ 83%), thereby confirming the fading‑memory dependence of the UCNC reservoir.


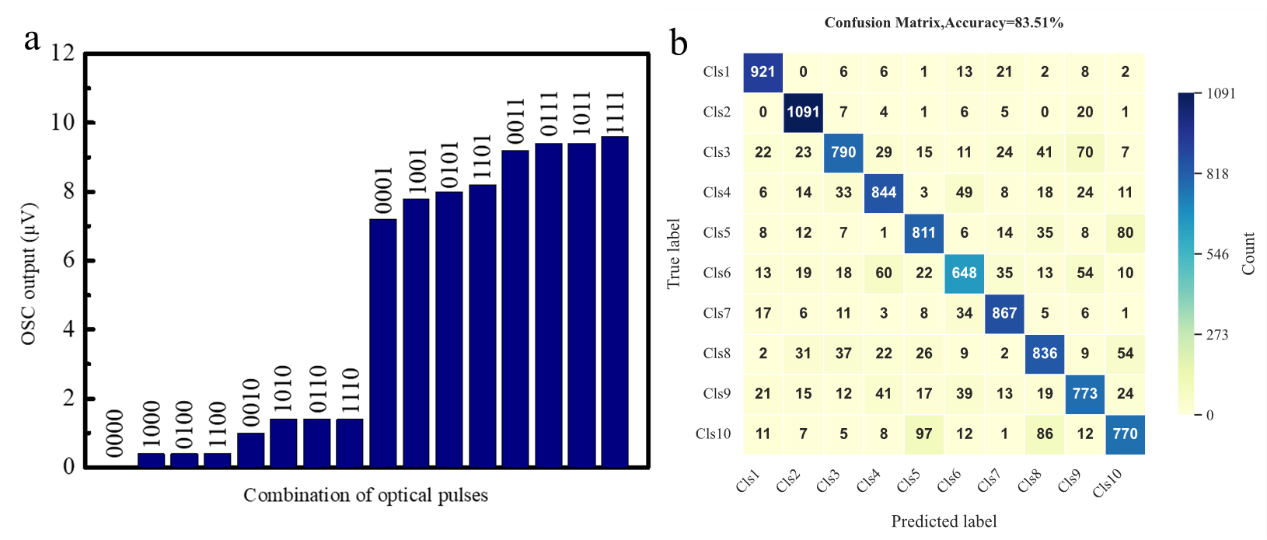


Figure S14. (a) Output response and (b) confusion matrix of the system without time dependence.

In the main text's time-series prediction section, we describe encoding the masked Mackey-Glass sequence onto an arbitrary waveform generator (AWG), where the information is modulated onto the injection light intensity. Here, we systematically evaluate the prediction performance across varying pulse periods. The results demonstrate consistently low normalized root-mean-square error (NRMSE) within the 25–100 μs range, beyond which the NRMSE exhibits gradual degradation. Thus, a pulse period of 25 μs is identified as the optimal trade-off between processing speed and prediction accuracy.

Figure S15. NRMSE vs pulse period.

Figure S16 presents more results from simulations and experiments that have been fitted (R² > 0.98 for all curves). The experimental measurement results have confirmed that the solutions of these dynamic equations exhibit excellent consistency with the actual output responses of the system.


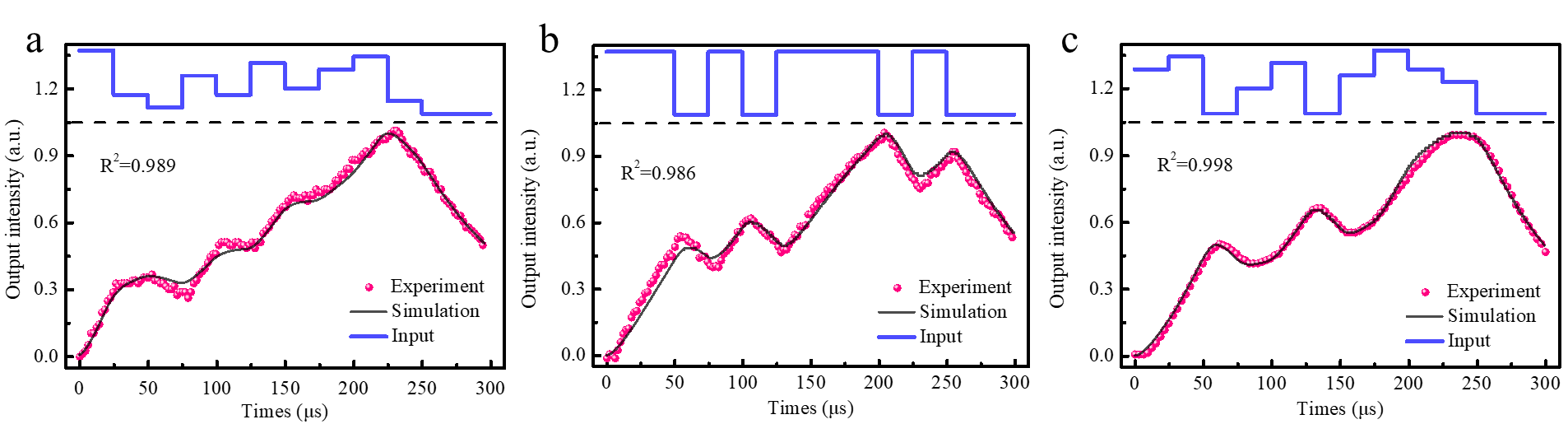


Figure S16. Multi-pulse response

In the time-series prediction section of the main text, we have demonstrated the capability of rare-earth-based reservoir computing in handling chaotic time-series prediction tasks. Our core approach involves utilizing the multi-level energy system of rare-earth ions as self-contained state units capable of autonomously performing reservoir layer computations, thereby proving the potential of rare-earth systems for all-optical information processing.

In this section, we establish more rigorous specifications for the input-output format by configuring the rare-earth system as dynamic nodes in a reservoir computing network, constructing a rare-earth reservoir neural network with echo state network architecture. The overall experimental setup remains largely consistent with that described in the main text. The key difference lies in the treatment of the output state vector from the rare-earth reservoir layer at each time step - this vector serves as a recurrent state variable that, together with the next input vector u(t+1), acts upon the rare-earth node. This architectural design endows the system with memory capacity for historical information.

Compared to the optical setup diagram in the main text, we have implemented a feedback connection between the system output and input sections, as illustrated in Fig. S17. This modification creates a closed-loop system that enables information recurrence while maintaining the same physical configuration of the rare-earth reservoir layer. The recurrent architecture allows each temporal state to incorporate both current inputs and prior system states, effectively implementing delay-based reservoir computing with the rare-earth material serving as the nonlinear processing medium.


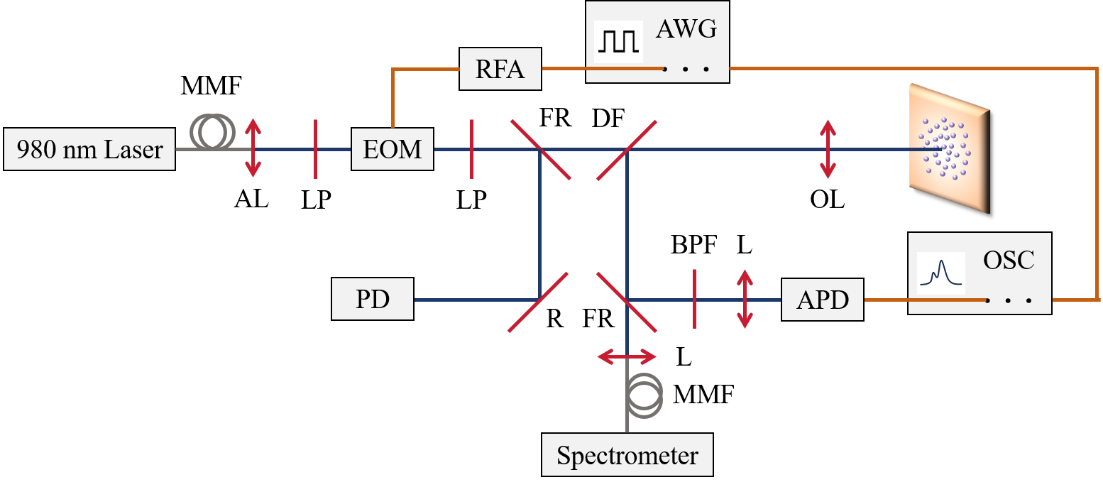


Figure S17. Modified Rare-Earth Reservoir Optical Path Diagram

Here, we continue to employ the Mackey-Glass equation to generate a one-dimensional time series spanning [1,3000] points, serving as the benchmark dataset for evaluating the temporal prediction capability of our rare-earth reservoir network.


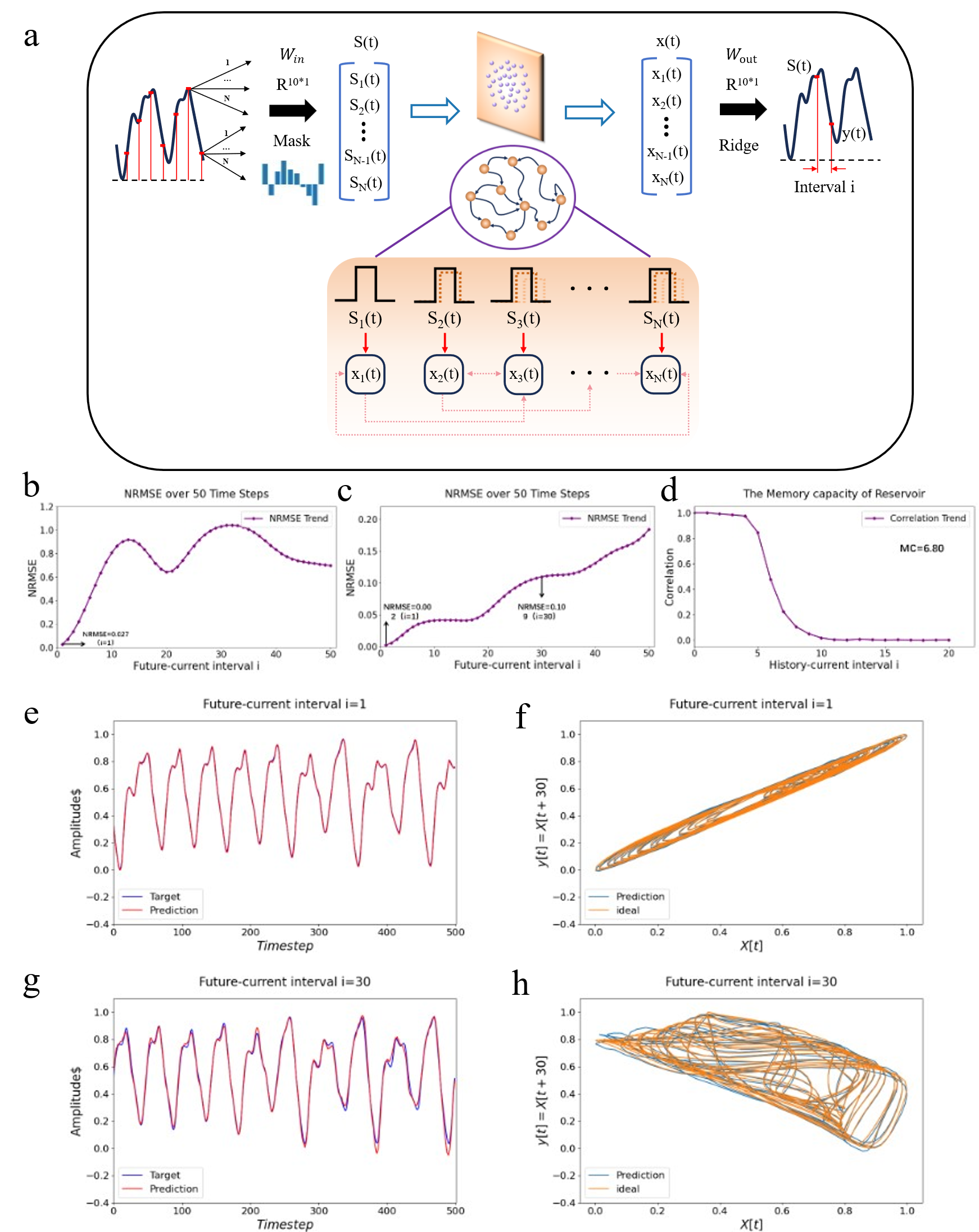


Figure S18. Mackey–Glass time-series prediction task. a. Schematic of the rare-earth reservoir. b. One-step-ahead prediction performance (n=1). c. 50-step-ahead prediction performance (n=50). d. Memory capacity of the rare-earth reservoir. e. Predicted vs. actual curves for n=1, i=1. f. Chaotic attractors of the predicted and actual sequences for n=1, i=1. g. Predicted vs. actual curves for n=50, i=30. h. Chaotic attractors of the predicted and actual sequences for n=50, i=30.

Here, following the methodology described in the main text, we encode the original Mackey-Glass sequence $\text{S}\text{(}\text{t}\text{)}$ into a row vector $\text{S}\text{(}\text{t}\text{)∈}\text{R}^{\text{1×}\text{L}}$and apply an N-row mask column vector $\text{W}_{\text{in}}\text{=}\left[ \text{w}_{\text{i}\text{1}}\text{ }\text{w}_{\text{i}\text{2}}\text{ …}\text{w}_{\text{iN}} \right]^{\text{T}}\text{∈}\text{R}^{\text{N}\text{×1}}$, where N corresponds to both the size of the mask matrix and the number of virtual nodes in the reservoir layer. Furthermore, we establish deterministic sparse connections between virtual nodes, with elements in the connection matrix following a random uniform distribution within the range$\text{[-0.5,0.5]}$. The weight coefficient matrix $\text{W}_{\text{r}}\text{∈}\text{R}^{\text{N}\text{×}\text{N}}$is constrained to satisfy the spectral radius condition, ensuring that the state variables of reservoir nodes remain stable during iteration without exhibiting rapid convergence or divergence. The connection weights are analyzed through an equivalent model of spatial distribution obtained via single-node time multiplexing. This architecture provides fundamental advantages: (1) hardware-efficient implementation through time-domain multiplexing, (2) guaranteed dynamical stability via spectral radius control, and (3) flexible trade-off between memory capacity and nonlinear processing through adjustable connection sparsity.

Since we aim to continuously record the state variables of the reservoir layer at each time step, we adopt the following implementation: After measuring the state variables of reservoir layer nodes at time t using an oscilloscope, we encode them into a vector format identical to that used at the next time step. This encoded vector is then concatenated with the input signal for time t+1 to form the composite input to the system. Consequently, at time t+1, the signal received by a given node in the reservoir layer can be expressed as:

$$\text{M}\text{(}\text{t}\text{+1)=}\left[ \begin{matrix} \text{X}\text{(t)} \\ \text{u}_{\text{i}}\text{(t+1)} \end{matrix} \right]$$

This signal is encoded by the AWG (Arbitrary Waveform Generator) into a continuous rectangular pulse train, where the magnitude of each element in the vector corresponds to the amplitude of individual rectangular pulses, while the weights mentioned above are mapped onto the pulse widths. Upon receiving the M(t+1) signal, each node generates its optical intensity response sequence X(t+1) according to the rate equations specified in the aforementioned formulation. This physical process corresponds to the experimental signal acquisition procedure performed by the photodetector and oscilloscope.

As demonstrated in Fig. S18(e, f), the system's single-step prediction results (n=1) are shown through comparative plots of predicted versus ideal Mackey-glass sequence evolution curves and corresponding chaotic attractors. Fig. S18(b) presents the Normalized Root Mean Square Error (NRMSE) evolution curve for multi-step prediction tests using only single-time-step reservoir node states. The NRMSE reaches 0.027 at τ=1 (characterizing the reservoir's fundamental prediction capability), but rapidly increases with larger τ values. In actual prediction plots (see Supplementary Information), the predicted curves exhibit localized high-frequency oscillations, indicating degraded prediction performance. When employing multi-time-step reservoir node states (n>1) for joint prediction, Fig. S18(c) reveals significant improvements: the system achieves both reduced overall NRMSE and enhanced multi-step prediction capability. For instance, at τ=5, the NRMSE=0.109, while the prediction curves and chaotic attractors shown in Fig. S18(g, h) closely approximate the ideal target sequence with smooth trajectories devoid of severe high-frequency artifacts, conclusively demonstrating the performance gains from using multiple reservoir states.

## **Note-5. Comparison between Re^3+^ UCNCs Based reservoir network and other physical RCs**

Finally, we compared our UCNCs-film-based based all optical RC and other physical RCs. As shown in Table S2. It can be seen that our work shows competitive performance in physical reservoir computing (RC) systems, particularly in terms of energy efficiency and processing speed. The UCNCs-film-based system achieves an energy consumption of ~μJ per bit, which compares favorably to prior photonic reservoir computing reports. This highlights the advantage of leveraging upconversion nanocrystals (UCNCs) for low-power, all-optical neuromorphic computing.

In terms of speed, our system processes input at 25 µs per bit, outperforming many electrical and optoelectronic devices (e.g., Fe-FEIs: 400 µs, Ti/NbOx/Pt: 10 ms) but slightly slower than ultrafast optical systems (e.g., QD-SA laser: 100 ps, Vcsel-MZM: 780 ps). This trade-off aligns with the inherent relaxation dynamics of rare-earth ions, which enable robust memory capacity at the cost of slower response times compared to semiconductor lasers.

For computational tasks, our RC system achieves NRMSE=8.4% in Mackey-Glass chaotic time-series prediction, comparable to QD-SA laser (NRMSE=8.1%) and superior to Vcsel-MZM (NRMSE=4.5%) in similar tasks. While not matching the accuracy of specialized ANN architectures (e.g., MNIST ACC=94% for Ti/NbOx/Pt), our approach excels in energy-efficient temporal signal processing, a critical requirement for edge-computing applications.

In conclusion, the advantage of our UCNCs-film-based RC lies in: (1).All-optical operation: Eliminates optoelectronic conversion losses, unlike hybrid systems (e.g., UCNPs@SiO2/P3HT); (2).Material-driven nonlinearity: UCNCs’ intrinsic dynamics simplify hardware design compared to engineered nonlinear components (e.g., MZM); (3). Scalability: Thin-film integration offers potential for compact, parallelized reservoirs. Future efforts could focus on accelerating relaxation times (e.g., via plasmonic enhancement) while preserving energy efficiency, potentially bridging the performance gap with ultrafast optical RC systems.

| Ref. | Device | Principle | I/O format | ISI | Energy  per bit | Architecture | Tasks and results |
| --- | --- | --- | --- | --- | --- | --- | --- |
| 1 | Perovskite TFTs | Carrier migration | O /E | 100ms | ~200pJ | Three-layer ANN | MNIST  (ACC=92.2%) |
| 2 | Fe-FETs | Phase transition | O /E | 400μs | 4.8aJ | RC+Cross-entropy function | MNIST  (ACC=91.7%)  Multiscale signal processing  (ACC=93.57%) |
| 3 | UCNPs@SiO2 /(P3HT) transistor | Carrier migration | O /El | 200ms | ~1nJ | RC+Cross-entropy function | MNIST  (ACC=91.13%) |
|  |  |  |  |  |  | RC+Ridge regression | Second-Order Nonlinear Dynamic Task  (NRMSE=3.3%) |
| 4 | QD-SA laser | Saturated absorption modulation | O /E | 100ps | 64aJ | RC+6‑fold cross‑validation | MIT-BIH arrhythmia detection  (ACC=98.4%)  4-class MNIST  (ACC=92.3%） |
|  |  |  |  |  |  | RC+Ridge regression | Mackey-Glass series prediction  (NRMSE=8.1%) |
| 5 | Ti/NbOx/Pt | Reversible resistance regulation | Electrical | 10ms | 89pJ | RC+Relu+Cross-entropy function | MNIST  (ACC=94%) |
| 6 | Vcsel-MZM | Nonlinear dynamics of VCSEL | Optical | 780ps | / | RC+Ridge regression | MNIST  (ACC=82%*)  Santa-Fe series prediction  (NRMSE=4.5%) |
| 7 | FP-SA | Saturated absorption modulation | Optical | 100ps | 7.329fJ | PSNN | / |
| This work | UCNCs-film | Nonlinear dynamics of UCNCs | Optical | 25μs | ~1 μJ | RC+Cross-entropy function | MNIST  (ACC=90.7%) |
|  |  |  |  |  |  | RC+Ridge regression | Mackey-Glass series prediction  (NRMSE=8.4%) |

Table S2. Comparison between UCNCs-film-based all optical RC and other physical RCs.

*Refers to the accuracy of binary masking.

**Reference:**

1. Liu Y, Lu Y, Yang X, et al. Amplified stimulated emission in upconversion nanoparticles for super-resolution nanoscopy, Nature, 2017, 543(7644): 229-233.
2. Y. Liu, S. Wen, F. Wang, C. Zuo, C. Chen, J. Zhou, D. Jin, Population Control of Upconversion Energy Transfer for Stimulation Emission Depletion Nanoscopy, Adv. Sci. 2023, 10, 2205990. <https://doi.org/10.1002/advs.202205990>.
